# Investigating combination HIV prevention: isolated interventions or complex system

**DOI:** 10.7448/IAS.18.1.20499

**Published:** 2015-12-14

**Authors:** Graham Brown, Daniel Reeders, Gary W. Dowsett, Jeanne Ellard, Marina Carman, Natalie Hendry, Jack Wallace

**Affiliations:** 1 Australian Research Centre in Sex, Health and Society, La Trobe University, Melbourne, Australia; 2 Centre for Social Research in Health, University of New South Wales, Sydney, Australia; 3 Kirby Institute, University of New South Wales, Sydney, Australia

**Keywords:** combination HIV prevention, evaluation, evidence, complex systems, intervention research, treatment as prevention

## Abstract

**Introduction:**

Treatment as prevention has mobilized new opportunities in preventing HIV transmission and has led to bold new UNAIDS targets in testing, treatment coverage and transmission reduction. These will require not only an increase in investment but also a deeper understanding of the dynamics of combining behavioural, biomedical and structural HIV prevention interventions. High-income countries are making substantial investments in combination HIV prevention, but is this investment leading to a deeper understanding of how to combine interventions? The combining of interventions involves complexity, with many strategies interacting with non-linear and multiplying rather than additive effects.

**Discussion:**

Drawing on a recent scoping study of the published research evidence in HIV prevention in high-income countries, this paper argues that there is a gap between the evidence currently available and the evidence needed to guide the achieving of these bold targets. The emphasis of HIV prevention intervention research continues to look at one intervention at a time in isolation from its interactions with other interventions, the community and the socio-political context of their implementation. To understand and evaluate the role of a combination of interventions, we need to understand not only what works, but in what circumstances, what role the parts need to play in their relationship with each other, when the combination needs to adapt and identify emergent effects of any resulting synergies. There is little development of evidence-based indicators on how interventions in combination should achieve that strategic advantage and synergy. This commentary discusses the implications of this ongoing situation for future research and the required investment in partnership. We suggest that systems science approaches, which are being increasingly applied in other areas of public health, could provide an expanded vocabulary and analytic tools for understanding these complex interactions, relationships and emergent effects.

**Conclusions:**

Relying on the current linear but disconnected approaches to intervention research and evidence we will miss the potential to achieve and understand system-level synergies. Given the challenges in sustaining public health and HIV prevention investment, meeting the bold UNAIDS targets that have been set is likely to be dependent on achieving systems level synergies.

## Introduction

In 2014, UNAIDS announced bold new targets for the global response to HIV (90% of people living with HIV (PLHIV) knowing their status, 90% of diagnosed PLHIV on treatment and 90% of PLHIV on treatment achieving an undetectable viral load) by 2020 [1]. In some high-income countries, similar ambitious targets have been set [2]. These goals follow from research that suggests HIV treatment can dramatically reduce transmission of HIV for PLHIV [3,4] and for people at risk of acquiring HIV [5]. These new developments have been described as “game changers” [6], adding new tools to a long-established mix of behavioural, biomedical and structural HIV prevention interventions. Achieving these goals will require not only an increase in HIV prevention and health system investment but also a deeper understanding of the dynamics of combining different HIV prevention interventions.

A partnership of affected communities, health services, government and research has been the foundation of many effective responses to HIV [7]. Although the scientific evidence for treatment as prevention is strong, achieving bold targets in testing, treatment coverage and transmission reduction will again rely on this partnership to achieve an integrated combination of strategies in the community sector, health services and policy environments. This combination of strategies will need to adapt as local epidemiology and social contexts shift and evolve in unpredictable ways.

The emergence of new prevention technologies and the recognized complexity of the treatment cascade [8] underscore the need for research into the different and complex combinations that may be required in local epidemics. Understanding how to achieve beneficial synergy among the interventions is a central and recognized challenge for combination prevention [9–11].

### Combination HIV prevention

Combination HIV prevention as a term emerged in the early 2000s but evolved further at the 2008 International AIDS Conference [11,12]. The concept draws on the term “combination” in combination antiretroviral therapy. Instead of prevention “monotherapy,” it proposed seeing HIV prevention as a combination of “potentially synergistic prevention activities” [10].

Although there is some disagreement on the definition of combination prevention, the key features in the UNAIDS discussion paper [13] included evidence-informed, simultaneous use of behavioural, biomedical and structural prevention strategies that are planned and managed to operate synergistically, and are flexible enough to permit continual adaptation to the changing environment. Central to most definitions is the combination of behavioural, biomedical and structural interventions – an imprecise shorthand for a wide range of HIV prevention interventions and services across categories with unclear boundaries. However, in general:

- Behavioural interventions are aimed at achieving changes in individual behaviour, such as use of condoms and safe injecting equipment, regular HIV testing or uptake of treatment (for prevention or health management). These include interventions such as peer education, community outreach, counselling and social marketing.
- Biomedical interventions are aimed at achieving improved prevention of HIV transmission through biomedical technology. These include, but are not limited to, the use of HIV medications for post- or pre-exposure prophylaxis (PEP, PrEP) or the achievement of undetectable viral load in a person with HIV.
- Structural interventions are aimed at influencing the social, political and institutional enablers, barriers and drivers of HIV epidemics. These include law reform; community leadership; access to health services, condoms and/or injecting equipment; reducing stigma or gender inequity; and increasing community resilience and political commitment. The focus is on promoting health by altering the structural context within which health is produced and reproduced [14].

### Additive or synergy

Although combination prevention is consistent with the foundations of the Ottawa Charter for Health Promotion [15] and has been presented as a “packaging” of complementary prevention interventions [10], a question arises. Is adding more and more ingredients to the mix, with little recognition of the need to craft a strategic mix, to adapt the mix over time or to respond to emerging consequences, simply an additive approach that cannot assess the synergies achieved in that packaging?

Most high-income countries are making substantial investments in combination HIV prevention, but will this investment lead to a deeper understanding of how to combine interventions? Will the current intervention research assist policymakers and communities to implement effective and adaptive combinations of HIV prevention interventions?

This paper argues that there continues to be a major gap between the evidence needed and the evidence currently available. We draw briefly on the results of a recent scoping study by Authors 1, 4 and 5 (see Table 1) to illustrate key themes in the current published evidence and then discuss the implications of this situation for future research and the partnerships this will require.

**Table 1:** Summary of intervention research scoping study

Scoping review question	What is the focus of published evidence regarding HIV prevention and health promotion interventions in high-income countries with concentrated epidemics?
Approach	Systematic scoping review as described by Arkey and O'Melley [17] in that it mapped the focus, rather than assessed the results, of the studies.
Data bases searched	EMBASE (Ovid), Informit Health, Medline, ProQuest, SAGE, SCOPUS (Elsevier), Web of Science [ISI], PsychInfo, Science Direct.
Search terms	HIV prevention, HIV health promotion and HIV combination prevention. These were coupled with terms such as review, evaluation, evidence, intervention, implementation, intervention focus (such as individual, group, community, structural), social drivers, programme theory, programme logic, systems.
Inclusion	Published in English between January 2006 and June 2013. Focused on or included analyses of HIV prevention and health promotion evidence and evaluation regarding sexual transmission of HIV in high-income countries with concentrated epidemics.
Exclusion	Exclusively laboratory-based biomedical and clinical studies. Focused exclusively on preventing HIV transmission through mother-to-child transmission, as these were rare occurrences in the Australian HIV epidemic. Focused exclusively on public health mechanisms not being proposed in Australia, such as male circumcision.
Published peer reviewed literature	The search yielded 2,598 papers. The titles of the papers were reviewed against the inclusion criteria and reduced to (522 papers). These papers were reviewed in detail and relevant papers were removed as per the exclusion criteria, if were duplicates, if were included in subsequently identified systematic reviews, or if they had been superseded by later papers. This resulted in 284 papers remaining.
Grey literature search	English language abstracts from key conferences where health promotion practice and intervention science was presented (such as the International AIDS Conference and key regional conferences in Europe, North America and Australasia). Reports and reviews from key government and non-government websites in Europe, North America and Australasia. This process added 212 reports, reviews and conference papers.
Mapping of literature	A total of 496 papers were included in the review. The papers were analyzed and mapped using the “level of intervention” categories adopted in the *Lancet* series [9] and UNAIDS technical guidance on combination prevention [13]. These include individual, group, behavioural, biomedical, community and structural levels.
Findings	Majority of research focus • Interventions aimed at individuals and small groups. Moderate research focus • Social marketing and community development in HIV prevention. • Underlying social and behavioural theories and quality practice. • How biomedical strategies may be effective in different contexts and among different populations outside trial conditions. Least research focus • Interventions that operate at or target the structural level. • Understanding the mechanisms and common factors within interventions that can be adapted. • Evaluation of the synergies within a combined HIV prevention system.
	The full report is available online [16]. www.latrobe.edu.au/arcshs/publications

## Discussion

### HIV prevention intervention research

In the lead-up to the development of Australia's Seventh National HIV Strategy [2], a scoping study of HIV intervention research conducted in high-income countries and published between 2006 and 2013 was undertaken to identify gaps and guide future evidence-building research [16]. The scoping study provided a useful overview of the extent to which recent published research evidence from high-income countries was responding to calls for a broader and more comprehensive evidence base to guide the combining of behavioural, biomedical and structural interventions. Summarized in Table 1, the findings of the scoping study mapped 496 publications using the “level of intervention” categories adopted in the *Lancet* series [9] and UNAIDS technical guidance on combination prevention [13]. These include individual, group, behavioural, biomedical, community and structural levels. The full methodology and findings report is available online [16]. From the findings of this scoping study we can draw three key themes about the evaluation of HIV prevention.

### Theme 1: A focus on individual behaviour change

It is well recognized that intervention research in HIV has focused on interventions targeting short-term individual behaviour change with limited attention given to researching the role of structural changes [18,19]. The scoping study found no evidence of a significant change in this emphasis. The literature continued to be dominated by experimental trials with a focus on intervention fidelity and aimed at controlling external or contextual variables to determine the contribution of the single intervention [20–22].

Experimental methodologies are preferred, and individual outcomes are methodologically simpler and logistically easier to study by these methodologies than broader structural interventions. This can reinforce a policy and funding focus on individual outcomes and, consequently, lead to research questions focused on individual outcomes. As argued by Coates et al. [10], the reliance on experimental designs to determine a suite of effective interventions can influence the type of interventions that are studied and therefore funded.

### Theme 2: Evaluating isolated interventions

The scoping study found the literature was dominated by intervention research that sought to measure the effect attributable to each intervention or programme in isolation, excluding effects attributable to interactions with other programmes or the local community and socio-political context. This approach struggles to model and measure the intersection of reciprocal or mutual influences (positive or negative) within a mix of interventions. There was little published research that attempted to evaluate the influences, synergies and conflicts between interventions within an overall combination approach, or which tracked the combination over time to identify any changes required as epidemiological, behavioural and structural contexts underwent changes of their own. The literature recognized that the impact of individual interventions may have been influenced by how they interacted with, and evolved in, a local context, referring to variables and interactions that an experimental study normally aims to control [23,24]. Nevertheless, most of the literature made little contribution to understanding whether a combination of interventions at a particular time and location was more or less effective than the sum of its parts in the same circumstances. The current research focus adds to an evidence base useful for decisions about single interventions, but treating potential synergies as confounders is less helpful for a strategic combination of interventions.

### Theme 3: Limited implementation experience

The scoping study found that most research reflected an assumption that the interventions being tested would be implemented as a new intervention; little research presented evidence on how to adapt existing interventions to maintain or improve their effectiveness over time. Few offered clear explanations of the mechanisms that produce outcomes in context, or guidance on what mechanisms need to be preserved when interventions were adapted in different settings [25–27]. Programmes that focused on disseminating evidence-based HIV prevention packages (derived from experimental trials) across the HIV sector described challenges in their dissemination because of the need to adapt the packages to ensure effectiveness in local contexts with different social structures and health services [28], or respond to fundamental changes such as the role of treatment in prevention [29].

### Implications

HIV has long been recognized as having its causes and consequences “deeply embedded in social, cultural and political processes” [30] and the response has always included adapting to changing epidemiological, technological and community developments [7,18]. However, the emphasis of HIV prevention intervention research in high-income countries continues to look at one intervention at a time (predominantly focused on individual behaviour), in isolation from its interactions with other interventions, and the community and the socio-political context of their implementation. When interventions are researched as isolated activities, this may reinforce the perspective within policy and funding agencies that interventions operate in isolation and their combined influence is simply additive and linear. This perspective directs attention away from identifying the relationships between interventions that could enhance impact or result in unintended negative consequences. It also supports the assumption that the only adaptation to “proven” interventions are tailoring and fine-tuning, rather than actively adapting and reorientating to changes produced by any number of forces, such as the environment, health system supply chain problems, political and funding changes, and many others.

### Combination prevention as a complex system

What is needed is research that recognizes that interventions and the systems of which they are part can be complex, dynamic, fluid and can be pressured or resistant to change. As has been argued previously [31–34], we require research and evaluation approaches that are focused on understanding the relationship between different interventions as well as between interventions and their environment. This means recognizing combination prevention as a “complex system.”

Complex systems are made up of heterogeneous elements that interact with one another and produce effects that are different from the effects of individual elements. These effects are emergent and not easily predictable and will adapt to changing circumstances [35]. Approaching combination prevention as part of a complex system asks us to consider the multiplying and amplifying effects of the relationship between elements in a system and its emergent overall effects. It helps us recognize that the way communities respond, enhance, adapt, resist or ignore interventions are part of the intervention process itself and not just confounders to the implementation of a predeveloped intervention. For example, the introduction of PrEP has highlighted the complexity inherent in combination HIV prevention [36,37]. PrEP has reciprocal interactions with health systems; community understandings of safe sex, HIV stigma, homophobia and moralism about sexual behaviour; and health literacy disparities in ways that cannot be easily predicted. This influence began before PrEP was more widely available in the United States [38] and is already occurring in other high-income countries where access is limited to importing from overseas [39]. PrEP has the capacity simultaneously to increase judgement and stigma about sexual behaviour and to decrease fear and stigma in sexual encounters. The system in which PrEP is to be incorporated into a combination prevention approach is a rapidly changing environment.

Combining interventions means recognizing that components in the system will interact and influence each other whether this is planned or not. The emphasis of combination prevention should be to gain the best strategic advantage and synergy from that interaction as it adapts and evolves. At present, however, there is little development of good quality evidence-based indicators on how interventions in combination work and how they should be funded, developed, implemented, evaluated and adapted to achieve the strategic advantage and synergy hoped for.

Systems science is an emerging approach in public health that has seen substantial uptake and application in other complex health and social challenges such as obesity [40–43], tobacco [44], and other areas [45]. Systems science approaches are a collection of analytic tools, such as system dynamics, network analysis, and agent-based modelling, that aim to examine simultaneously the big picture, the individual pieces that make up the picture and the complexity of non-linear relationships and emergent effects [35,46,47]. As argued by Skinner and colleagues [43, p. 2] in their work in obesity prevention:

Systems science offers a means of identifying and understanding the complex relationships involved in public health policies. It recognizes that policies are based on complex, interdependent and evolving relationships and include heterogeneous agents (e.g., individuals, companies or civic associations) acting in their own perceived self-interests. Time matters, as relationships among the agents have a history and, as a result, can develop stability or even inertia. In a complex system, intervention in one aspect will have unanticipated effects, often delayed and non-linear. Such effects are not exceptions but the norm.

There have been substantial investments into the evaluation of large-scale combination prevention programmes in some low-income countries with generalized epidemics [48–50] as well as developments in the use of implementation and operations research [51]. However, most investments have not focused on the relationships between the components of a combination prevention system or the ongoing adaptations required because of unpredictable interactions and dynamics. Without a deeper understanding of combination prevention dynamics in concentrated epidemics and high-income countries, it is difficult to translate or adapt the findings we do have from one country to another, particularly when the contexts are so different.

There have been few applications of systems thinking in HIV prevention, despite its potential contribution to understanding combination HIV prevention. Some emerging examples include the application of complex adaptive systems theory in initiatives like the “What Works and Why” project (www.w3project.org.au) in Australia that is looking at the behavioural and structural influence of community-led programmes, the application of continuous adaptation and quality improvement in initiatives like European Quality Action (www.qualityaction.eu) in Germany and the structural intervention modelling focus of projects like STRIVE (strive.lshtm.ac.uk) in the United Kingdom. Drawing on systems science to understand combination prevention as a complex system may be an approach to bring clarity to the relationships between independent HIV interventions.

### Partnership

Evaluating combination prevention as a complex system, however, significantly increases the challenge for research and evaluation. These are not limited to debates about epistemology and methodology. The challenge is equally, perhaps mostly, about political and policy courage to invest in a range of approaches, engagement with long-term emergent outcomes and the sharing of real time evaluation and strategic insights to guide ongoing adaptation. When evidence is focused on interventions in isolation, it can discourage and weaken the motivation for partnerships across agencies and encourage research to search for the single most effective intervention or the one most easy to measure. For example, achieving synergy between strategies on PrEP in a clinic and in the community may enhance the impact of both. However, evaluating these strategies in isolation where impact needs to be attributed to a single intervention can undermine a collaborative and synergistic approach.

Although building evidence is critical to understanding a complex system, such evidence will not automatically be shared or translated into policy and practice. This requires sharing and synthesising of evidence from many sources, as well the capacity and policy environment to take action when evidence is limited [7]. These approaches require not only significant investment of funds, but significant investment in partnership across disciplines, organizations and funding mechanisms. Implementing and evaluating combination prevention with a systems perspective will require cooperation among community organizations, health services, public health, law enforcement, researchers and clinicians – something the HIV response has previously achieved [7].

## Conclusions

Despite the increasing complexity in the HIV landscape and calls for intervention research to broaden its view, there is as yet little evidence of a substantial change in the focus of intervention research. The evidence to guide combination HIV prevention needs to move beyond measuring effects of interventions in isolation and incorporate methods that focus on the interactions between interventions, contexts and the emergent effects of systems that are not visible when viewing only its individual components. We need to understand how to ensure the quality and effectiveness of each intervention is mutually reinforcing, and how the combination should continuously adapt to changes in behavioural, biomedical and structural contexts.

Systems approaches may provide the expanded vocabulary for describing these complex non-linear interactions, relationships and emergent effects. However, this will also require a major investment in partnerships and commitment to openness. If we rely exclusively on the current dominant approaches and focus of intervention research and evidence, we will be guiding combined prevention programmes through the narrow lens of programmes in isolation, and missing the system-level synergies. Given the challenges in sustaining public health and HIV prevention investment, meeting the bold UNAIDS targets that have been set is likely to be highly dependent on achieving system-level synergies.
